# Congenital Amegakaryocytic Thrombocytopenia Presenting With Features of Immune Thrombocytopenia: A Case Report

**DOI:** 10.7759/cureus.102147

**Published:** 2026-01-23

**Authors:** Afsheen Umer, Shifa Nafis, Mehran Karimi

**Affiliations:** 1 Pediatrics, American Hospital Dubai, Dubai, ARE; 2 Pediatric Hematology and Oncology, American Hospital Dubai, Dubai, ARE

**Keywords:** bone marrow failure, bone marrow failure syndrome, congenital thrombocytopenia, hematology disorders, immune thrombocytopenia (itp), isolated thrombocytopenia, mpl gene, pancytopenia, pediatric rare disease, stem cell transplant

## Abstract

Congenital amegakaryocytic thrombocytopenia (CAMT) is a rare inherited bone marrow failure disorder characterized by severe thrombocytopenia due to absent or markedly decreased megakaryocytes. It typically presents in infancy and may initially mimic more common causes of thrombocytopenia, such as immune thrombocytopenic purpura (ITP), leading to delays in diagnosis. We describe the case of a young child who initially presented with isolated thrombocytopenia in early infancy and was treated as ITP. Despite multiple courses of standard ITP therapy, including corticosteroids and intravenous immunoglobulin, her platelet counts failed to improve. Over time, the development of progressive pancytopenia raised concern for an underlying bone marrow failure syndrome. Although bone marrow examination demonstrated hypocellularity, a definitive diagnosis of CAMT could not be established based on morphology alone. The diagnosis was ultimately confirmed by whole-exome sequencing, which identified a pathogenic mutation in the myeloproliferative leukemia virus oncogene (MPL) gene. This case highlights the diagnostic challenges posed by rare inherited thrombocytopenias that clinically resemble ITP. Most importantly, it underscores the limitations of bone marrow biopsy as a standalone diagnostic tool and emphasizes the critical role of genetic testing in establishing a definitive diagnosis when clinical suspicion persists. Early identification of CAMT improves long-term outcomes, prevents prolonged ineffective treatment, and facilitates timely referral for hematopoietic stem cell transplantation.

## Introduction

The most common cause of congenital amegakaryocytic thrombocytopenia (CAMT), a rare autosomal recessive bone marrow failure condition, is mutations in the thrombopoietin receptor gene (myeloproliferative leukemia virus oncogene (MPL)) [1]. Mutations in the thrombopoietin gene (THPO) have been documented less frequently. The condition usually manifests as isolated thrombocytopenia in infancy or early childhood, frequently resembling immune thrombocytopenic purpura (ITP) [1,2].

In contrast to ITP, CAMT is unresponsive to standard treatments such as intravenous immunoglobulin and corticosteroids. Widespread bone marrow failure may lead to pancytopenia if treatment is not provided. Worldwide, fewer than one hundred cases of this condition have been reported, with a slight predominance in females and a potential association with consanguineous relationships [3].

Clinically, affected infants frequently exhibit severe thrombocytopenia during the first few months of life, which is followed by a gradual decrease in red and white blood cell counts, placing patients at risk for potentially fatal bleeding and infections. Although bone marrow examination typically shows absent or significantly diminished megakaryocytes, non-specific or unclear findings may be observed in cases of early or evolving illness. Consequently, a reliable diagnosis now depends on genetic confirmation of MPL mutations [3,4]. The only effective treatment for CAMT remains allogeneic hematopoietic stem cell transplantation (HSCT). Early transplantation, before the onset of severe pancytopenia or clonal evolution, greatly improves outcomes [4,5].

## Case presentation

A 15-year-old girl first exhibited thrombocytopenia at three months of age, with a platelet count of approximately 100 × 10³/µL. She was not closely followed until the age of three years, when a repeat platelet count was obtained after an episode of melena. At that time, her hemoglobin and white blood cell counts were within normal limits, but she had marked thrombocytopenia (20 × 10³/µL). She was subsequently diagnosed with immune thrombocytopenic purpura (ITP) and treated with multiple courses of intravenous immunoglobulin and corticosteroids, without sustained improvement in platelet counts.

At six years of age, in the setting of persistent thrombocytopenia and evolving cytopenias, bone marrow aspiration and biopsy were performed. Laboratory evaluation at that time demonstrated anemia, severe thrombocytopenia, and marked neutropenia, with differential counts showing relative lymphocytosis, as summarized in Table 1. Given the lack of sustained response to standard ITP-directed therapies and the progression of cytopenias, further diagnostic evaluation, including genetic testing, was pursued.

**Table 1:** Hematologic parameters of the patient at six years of age.

Parameter	Result	Reference range
Red blood cell count (RBC)	2.38 × 10^12^/L	4.0–5.2 x 10^12^/L
Hemoglobin	9 g/dL	11.5–15.5 g/dL
Platelet count	7 × 10^9^/L	150–410 x 10^9^/L
White blood cells (WBC)	4.2 × 10^9^/L	4–11 x 10^9^/L
Neutrophils	21.2%	30–60%
Absolute neutrophil count	0.7 × 10^3^/µL	1.5–8 × 10^3^/µL
Lymphocytes	74.2%	35–55%
Absolute lymphocyte count	2.44 × 10³/µL	1.5–7 × 10^3^/µL

Her past medical history is otherwise unremarkable and includes delivery by cesarean section, an uncomplicated postnatal course, and up-to-date immunizations. Family history revealed healthy parents who are first-degree cousins, a healthy younger brother, and a healthy younger sister who served as the bone marrow donor. There was no known family history of congenital thrombocytopenia or bone marrow failure syndromes. Parental testing and formal genetic counseling were recommended to confirm homozygosity of the detected variant and to address recurrence risk.

On presentation, the patient was well-appearing and in no acute distress. Vital signs were within normal limits. Mental status was normal. The head, eyes, ears, nose, and throat (HEENT) examination was unremarkable, with no evidence of oral or wet purpura. Cardiovascular and respiratory examinations were normal, without murmurs or adventitious lung sounds. Abdominal examination revealed no hepatosplenomegaly. Neurologic, musculoskeletal, and lymphatic examinations were normal. The skin was intact, with no petechiae or purpura observed.

Peripheral blood smear evaluation demonstrated severely decreased platelets, mild macrocytosis, anisocytosis, and poikilocytosis, without abnormal inclusions, as detailed in Table 2. These findings supported an underlying marrow production defect rather than peripheral platelet destruction.

**Table 2:** Peripheral blood smear at six years of age. RBC: red blood cell count.

Feature	Finding
Platelets	Severely decreased
RBC morphology	Mild macrocytosis (1+), anisocytosis, poikilocytosis (1+)
Other	No abnormal inclusions reported

Bone marrow biopsy revealed decreased cellularity, consistent with bone marrow failure but not diagnostic of a specific inherited syndrome (Table 3). Infectious causes of cytopenias were investigated, and serologic testing for Parvovirus B19 showed negative IgG and IgM antibodies, excluding both prior and acute infection (Table 4).

**Table 3:** Bone marrow biopsy at six years of age.

Feature	Finding
Cellular density	Decreased cellularity
Interpretation	Consistent with marrow failure, not definitive for a specific inherited syndrome.

**Table 4:** Parvovirus B19 serology.

Test	Result	Reference range	Clinical note
Parvovirus B19 IgG	<0.5	Negative: <0.8; Borderline: 0.8–1.1; Positive: >1.1	No evidence of prior infection
Parvovirus B19 IgM	<0.5	Negative: <0.7; Borderline: 0.7–1.2; Positive: >1.2	No evidence of acute infection

Given the lack of response to standard ITP-directed therapies and the evolving marrow failure, whole-exome sequencing was pursued. Genetic analysis identified a homozygous missense variant in the MPL gene (NM_005373.2:c.382G>T; p.Asp128Tyr), classified as likely pathogenic (American College of Medical Genetics and Genomics (ACMG) class 2), confirming the diagnosis of congenital amegakaryocytic thrombocytopenia. Detailed variant characteristics, in silico predictions, and population frequency data are summarized in Table 5.

**Table 5:** Genetic variant identified on whole-exome sequencing. ESP: Exome Sequencing Project, SIFT: Sorting Intolerant from Tolerant, MPL: myeloproliferative leukemia virus oncogene, GVGD: Grantham Variation–Grantham Deviation.

Feature	Result	Interpretation/notes
Gene	MPL	Associated with congenital amegakaryocytic thrombocytopenia
Variant (cDNA/protein)	NM_005373.2:c.382G>T, p.(Asp128Tyr)	Missense variant changing aspartic acid to tyrosine at position 128
Zygosity	Homozygous	Both alleles carry the variant
In silico predictions	PolyPhen: Probably damaging; align-GVGD: C35; SIFT: –; MutationTaster: disease-causing; Conservation_nt: moderate; Conservation_aa: high	Computational evidence suggests functional impact
Population frequency	gnomAD: 0.000012; ESP: –; 1000G: 0.00020; CentoMD: –	Extremely rare, supporting pathogenicity
Variant type and classification	Missense; likely pathogenic (class 2)	Strong evidence for disease causation

From July 2021 to May 2022, the patient was treated with eltrombopag, a thrombopoietin receptor agonist, at a dose of 50 mg daily, resulting in minimal improvement in platelet counts. In the setting of progressive pancytopenia due to bone marrow failure, she subsequently underwent matched sibling donor allogeneic hematopoietic stem cell transplantation. Post-transplant chimerism analysis demonstrated 96% donor cells initially, increasing to 98% at the most recent follow-up. Immunosuppressive medications, including cyclosporine and acyclovir, were discontinued per protocol.

Following transplantation, the patient demonstrated sustained clinical and hematologic recovery. Recent complete blood count parameters showed normalization of white blood cell count, hemoglobin, and platelet levels, as outlined in Table 6. Renal function and electrolyte measurements remained within normal limits (Table 7), and liver function tests were also normal (Table 8), indicating preserved organ function post-transplant.

**Table 6:** Recent complete blood count. Post-transplant hematologic evaluation shows normalization of white blood cells, hemoglobin, and platelets, reflecting sustained recovery following matched sibling donor hematopoietic stem cell transplantation.

Parameter	Result	Reference range
White blood cells (WBC)	4,900/µL	4000–11,000/µL
Hemoglobin (Hb)	13.3 g/dL	11.5–15.5 g/dL
Platelets	263 × 10³/µL	150–410 × 10³/µL

**Table 7:** Recent renal profile and electrolytes. Renal function and electrolyte measurements are within normal limits, indicating preserved kidney function and homeostasis after transplantation.

Parameter	Result	Reference range
Glucose	5.5 mmol/L	3.9–6.0 mmol/L
Urea	4.6 mmol/L	0–8.3 mmol/L
Creatinine	30 µmol/L	29–56 µmol/L
Sodium	141 mmol/L	136–146 mmol/L
Potassium	4.1 mmol/L	3.5–5.1 mmol/L
Chloride	106 mmol/L	98–107 mmol/L
Bicarbonate	23.6 mmol/L	22–32 mmol/L
Inorganic phosphate	1.26 mmol/L	1.05–1.7 mmol/L

**Table 8:** Recent liver profile. Liver function tests, including protein, bilirubin, transaminases, and alkaline phosphatase, are within normal ranges, consistent with normal hepatic function post-transplant. ALT: alanine aminotransferase, ALP: alkaline phosphatase, AST: aspartate aminotransferase.

Parameter	Result	Reference range
Total protein	71 g/L	64–83 g/L
Albumin	44 g/L	38–54 g/L
Total bilirubin	7.0 µmol/L	0–21 µmol/L
Direct bilirubin	3.2 µmol/L	0–7 µmol/L
ALP	219 U/L	129–417 U/L
ALT	9 U/L	0–33 U/L
AST	11 U/L	0–32 U/L

A chronological summary of the patient's clinical course, diagnostic evaluations, therapeutic interventions, and outcomes is presented in Table 9, illustrating the progression from early thrombocytopenia initially misdiagnosed as ITP to the definitive genetic diagnosis of CAMT and successful curative transplantation.

**Table 9:** Clinical timeline of the patient with congenital amegakaryocytic thrombocytopenia. IVIG: intravenous immunoglobulin, MPL: myeloproliferative leukemia virus oncogene, ITP: immune thrombocytopenic purpura, HSCT: hematopoietic stem cell transplantation, CAMT: congenital amegakaryocytic thrombocytopenia, WBC: white blood cells.

Age/time point	Clinical event	Key findings	Management/outcome
Three months	Initial presentation	Thrombocytopenia (100 × 10^3^/µL)	No specific follow-up
Three years	Episode of melena	Severe thrombocytopenia (20 × 10^3^/µL); normal Hb and WBC	Diagnosed as ITP; IVIG and corticosteroids
Three to six years	Persistent disease	No sustained platelet response	Continued ITP-directed therapy
Six years	Worsening cytopenias with diagnostic evaluation, peripheral smear showing severe thrombocytopenia and mild macrocytosis, Parvovirus B19 serology negative.	Bone marrow aspiration and biopsy with further evaluation	Decreased cellularity with non-specific findings
Six years	Genetic testing	Homozygous pathogenic MPL variant	Diagnosis of CAMT confirmed
Post-therapy	Progressive pancytopenia due to marrow failure	Matched sibling donor allogeneic HSCT	Definitive therapy
Follow-up	Post-transplant course	Normalization of blood counts	Sustained hematologic remission

## Discussion

Congenital amegakaryocytic thrombocytopenia is a rare inherited disorder characterized by severe thrombocytopenia due to absent or markedly reduced megakaryocytes in the bone marrow. Its early presentation often closely resembles ITP, which can result in misdiagnosis and prolonged exposure to ineffective therapies. Persistent thrombocytopenia unresponsive to corticosteroids or intravenous immunoglobulin should prompt reconsideration of the diagnosis and evaluation for inherited bone marrow failure syndromes [5].

A classification system based on disease course and outcome was proposed in 2005 and supported by subsequent reports. Type I CAMT is characterized by early-onset severe pancytopenia, markedly reduced bone marrow activity, and profound thrombocytopenia, typically resulting from complete loss of functional c-MPL receptor activity. Patients in this group usually have median platelet counts of ≤21 × 10^9^/L and experience rapid progression to bone marrow failure. Type II CAMT represents a milder phenotype, with transient increases in platelet counts--sometimes approaching near-normal levels--during the first year of life, followed by development of bone marrow failure between three and six years of age or later. In this group, partially functional c-MPL receptors are present, and median platelet counts generally range from 35 × 10^9^/L to 132 × 10^9^/L. Type III CAMT is characterized by ineffective megakaryopoiesis without detectable defects in the c-MPL gene [5,6].

The clinical course of our patient is most consistent with Type II CAMT, given the initial presentation with isolated thrombocytopenia, transiently preserved hematopoiesis, and subsequent progression to pancytopenia in early childhood. Importantly, this case also illustrates the limitations of bone marrow examination alone, as early marrow findings may be non-specific or inconclusive. In our patient, a definitive diagnosis was achieved through molecular testing, with identification of a homozygous pathogenic MPL variant, underscoring the critical role of genetic analysis in suspected cases of CAMT.

Although bone marrow examination is traditionally considered central to the diagnosis of CAMT, this case illustrates its limitations. In early or evolving disease, bone marrow findings may be non-specific or inconclusive. In our patient, the diagnosis was ultimately established through genetic testing, with the identification of a pathogenic MPL mutation, emphasizing that molecular analysis is indispensable when clinical suspicion remains high despite inconclusive marrow findings.

The outcome for patients with CAMT declines markedly once pancytopenia develops. Mortality is often associated with complications from hematopoietic stem cell transplantation and bleeding events. Many affected children may develop aplastic anemia during infancy, while disorders such as myelodysplastic syndrome or leukemia can appear later in childhood [7,8].

Allogeneic hematopoietic stem cell transplantation remains the only curative treatment for congenital amegakaryocytic thrombocytopenia associated with c-MPL mutations. Early transplantation, preferably before the development of severe pancytopenia or clonal evolution, is associated with improved survival and reduced morbidity. Delaying transplantation increases the risks related to repeated transfusions, including alloimmunization and infection. Until definitive therapy is undertaken, optimal supportive care, including platelet transfusions, infection monitoring, and bleeding prevention, is essential. Comprehensive patient and caregiver education is crucial, with emphasis on early recognition of complications and avoidance of activities that increase bleeding risk [9].

## Conclusions

Congenital amegakaryocytic thrombocytopenia (CAMT) is an uncommon but serious disorder that can mimic more common causes of thrombocytopenia in children, such as ITP. This case highlights the importance of maintaining a high index of suspicion in children with persistent, treatment-resistant thrombocytopenia, particularly in the context of consanguinity. Genetic testing is essential for definitive diagnosis, even when a bone marrow biopsy is performed, and early confirmation prevents unnecessary and ineffective treatments, improves outcomes, and enables timely referral for hematopoietic stem cell transplantation (HSCT). Allogeneic HSCT remains the only curative treatment for CAMT due to MPL mutations and is most effective when performed before severe pancytopenia develops.

The prognosis is poor once pancytopenia occurs, with patients at risk for life-threatening bleeding, HSCT-related complications, early aplastic anemia, and, in some cases, later development of leukemia or myelodysplasia. Timely hematopoietic stem cell transplantation (HSCT) can lead to sustained normalization of blood counts, long-term hematologic remission, and improved survival, though ongoing follow-up is necessary to monitor for late complications such as graft-versus-host disease, infections, or organ dysfunction. Additionally, genetic counseling is critical for families with consanguinity, providing guidance on recurrence risk, early screening of siblings or future pregnancies, and preventive care planning. Overall, early recognition, definitive genetic diagnosis, and timely HSCT offer affected children the best chance for long-term disease-free survival and good quality of life.
